# Liver function in dengue and its correlation with disease severity: a retrospective cross-sectional observational study in a tertiary care center in Coastal India

**DOI:** 10.11604/pamj.2021.40.261.29795

**Published:** 2021-12-23

**Authors:** Anusha Mruthyunjaya Swamy, Prasanth Yelkana Mahesh, Sujith Tumkur Rajashekar

**Affiliations:** 1 Department of General Medicine, Father Muller Medical College and Hospital, Mangalore-575002, India,; 2 Star Academy of Statistics, Bangalore-560070, India

**Keywords:** Dengue, liver, serum albumin, bilirubin, transaminases

## Abstract

**Introduction:**

dengue, the most important arthropod- borne disease is transmitted to humans by mosquitos of the Aedes family. Liver dysfunction in dengue varies from mild injury with elevation of transaminases to severe hepatocyte injury. The aim of our study was to assess the prevalence of hepatic dysfunction in patients with dengue and to correlate between the severity of the disease with the extent of hepatic dysfunction.

**Methods:**

retrospective cross-sectional observational study including 120 patients with confirmed dengue serology admitted in Medicine Department of Father Muller Medical College during November 2018-December 2019. Patient demographics, presenting symptoms, clinical signs, laboratory parameters such as complete blood count, serum glutamic-oxaloacetic transaminase (SGOT), serum glutamic-pyruvic transaminase (SGPT), alkaline Phosphatase (ALP), total and direct bilirubin; serum albumin and globulin levels were collected. Patients were categorized based on the modified WHO classification of 2009 into dengue with or without the warning signs and severe dengue. Comparison of multiple means across disease severity was performed using One Way-ANOVA with post hoc analysis using least significant difference. Pearson's correlation coefficient test was used to calculate the correlation between transaminases and platelet count. P-value <0.05 and CI 95% were considered in all analyses.

**Results:**

serum glutamic-oxaloacetic transaminase was elevated in 66.7%, 78.6% and 91.7% patients of dengue without warning signs, warning signs and severe dengue respectively. Serum glutamic-pyruvic transaminase was elevated in 42.4%, 52.4% and 91.7% patients of dengue without warning signs, warning signs and severe dengue respectively. Patients with elevated SGOT (93.8%) and SGPT (81.2%) had a higher incidence of bleeding manifestations. Hypoalbuminemia (50.8%) and A: G ratio reversal (27.3%) was significantly more in severe dengue (p<0.0001). Serum glutamic-oxaloacetic transaminase and serum glutamic-pyruvic transaminase levels negatively correlated with platelet count (p<0.0001).

**Conclusion:**

liver involvement in the form of elevated transaminases was found in 74.2% dengue patients. Serum glutamic-oxaloacetic transaminase and serum glutamic-pyruvic transaminase level increases with increase in dengue severity which is indicated by fall in platelet count as they are negatively correlated with each other. Liver damage is one of the common complications of dengue and transaminitis, hypoalbuminemia and reversal of A: G ratio should be used as biochemical markers in dengue patients to detect and monitor hepatic dysfunction.

## Introduction

Dengue virus (DENV), the most important arthropod- borne disease is transmitted to humans by mosquitos of the *Aedes* family [1]. All four dengue virus serotypes (DENV-1, DENV-2, DENV-3 and DENV-4) can cause the disease which can present as a mild self-limiting illness, dengue fever (DF), or as the more severe forms of the disease, dengue hemorrhagic fever (DHF) or dengue shock syndrome (DSS) [2]. The modified categorization of WHO in 2009 includes dengue with or without warning signs or severe dengue [3]. In spite of the recent categorization, the majority of the studies widely use the more popular DF, DHF and DSS classification for case definition.

Dengue virus infection is a public health problem in tropical and subtropical regions of the world. In India, dengue is endemic in almost all States and is the leading cause of hospitalization [4]. The disease had a predominant urban distribution a few decades earlier but is now also reported from peri-urban as well as rural areas [5,6]. During 2019, the National Vector Borne Disease Control Program reported more than 1.5 lakh laboratory confirmed cases of dengue [7]. It is therefore possible that dengue disease burden is grossly under-estimated in India.

Hepatic injury with dengue infection has been described since 1967 [8]. Liver dysfunction varies from mild injury with elevation of transaminases to severe hepatocyte injury, resulting in jaundice. Direct hepatotoxicity as well as deranged host immune response against the virus is responsible for the hepatic dysfunction. Though there have been isolated cases of fulminant hepatic failure, the derangements in the transaminases are usually self-limiting and may serve as a predictor for assessing the disease severity [9,10]. Limited studies are available in our geographic location to understand the pattern of liver involvement in dengue patients based on 2009 WHO categorization. We have sought to address this gap in the literature by conducting a study in coastal Indian population. Our aim of the study was to assess the prevalence of hepatic dysfunction in patients with dengue and to correlate between the severity of the disease with the extent of hepatic dysfunction.

## Methods

This was a retrospective observational cross-sectional study including 120 patients with confirmed dengue serology admitted in Medicine Department of Father Muller Medical College and Hospital during November 2018 - December 2019. The present study was conducted after obtaining approval from the institutional ethics committee and in accordance with ICH-GCP guidelines. Patients above 18 years of age with confirmed dengue serology (IgM positive by spot) were included in the study. Patients with chronic liver disease; history of long-term intake of hepatotoxic drugs; concurrent infections causing hepatitis- such as but not limited to leptospirosis, viral hepatitis, malaria, secondary sepsis; and patients with history of non-alcoholic fatty liver disease and non-alcoholic steato-hepatitis were excluded.

The patients were categorized based on the modified WHO classification of 2009 into dengue with or without the warning signs and severe dengue [3]. The following data was collected from the hospital medical records - patient demographics, severity of dengue, presenting symptoms, clinical signs, laboratory parameters - complete blood count, SGPT, SGOT, ALP, total and direct bilirubin; serum albumin and globulin levels. The sample size was calculated to be 120, assuming the anticipated prevalence of hepatic dysfunction in dengue to be around 50% [11] assuming α error 5% (Z_α_= 1.96) and β error 20% (Z_β_= 0.842) and a power of 80%, with a precision of 5%, according to the following formula:


$$n=\frac{\left[{\left(Z_{\alpha }+Z_{\beta }\right)}^{2}+pq\right]}{d^{2}}$$

Where p = prevalence; q= (1- p); and d = precision.

Data analysis was done using the Statistical Package for the Social Science (SPSS) Version 20. Continuous variables were summarized using mean and standard deviation (SD) and categorical variables as frequencies and percentages. Comparison of multiple means across disease severity was done using One Way-ANOVA with post hoc analysis using least significant difference. Pearson´s correlation coefficient test was used to calculate the correlation between variables (transaminases and platelet count). A two tailed probability value of <0.05 (95% CI) was accepted as the level of statistical significance.

## Results

Out of 120 patients, 66 patients (55%) had dengue without warning signs, 42 patients (35%) had dengue with warning signs and 12 patients (10%) had severe dengue. The mean age (SD) of the patients was 34.8 (15.1) years. More than 50% of the patients infected with dengue were aged between 18 and 30 years. Severe dengue was commonly seen in patient aged more than 40 years. Male to female ratio was 1.6: 1. Among the 12 patients with severe dengue, 5 were male patients and 7 were female patients. There was no statistically significant difference in patients´ age and gender distribution across dengue severity groups.

Serum glutamic-oxaloacetic transaminase was elevated in 66.7%, 78.6% and 91.7% of the patients of dengue without warning signs, with warning signs and severe dengue respectively. Serum glutamic-pyruvic transaminase was elevated in 42.4%, 52.4% and 91.7% of the patients of dengue without warning signs, with warning signs and severe dengue respectively. Alkaline phosphatase was elevated in 10.6%, 21.4% and 58.3% of the patients of dengue without warning signs, with warning signs and severe dengue respectively. Hyperbilirubinemia was seen in 1.5%, 16.7% and 25% of the patients in dengue without warning signs, with warning signs and severe dengue respectively. Also, 87.9%, 95.2% and 100% patients had a SGOT/SGPT ratio of >1 in dengue without warning signs, warning signs and severe dengue respectively. Hypoalbuminemia was found in 9.1%, 21.4% and 50% patients of dengue without warning signs, with warning signs and severe dengue respectively. Reversal of A: G ratio, that is A/G ratio <1 was seen in 1.6%, 7.3% and 27.3% of the patients of dengue without warning signs, warning signs and severe dengue respectively. Mean distribution of platelets, SGOT, SGPT, ALP, total bilirubin, direct bilirubin and serum albumin based on disease severity is represented in Table 1.

**Table 1:** liver function abnormalities in dengue

		Dengue classification			P-value (95% CI)		
		Dengue without warning signs (DWWS) (n=66)	Dengue with warning signs (DWS) (n=42)	Severe dengue (SDG)(n=12)	DWWS VS. DWS	DWWS VS. SDG	DWS VS. SDG
**Platelet count (cu.mm)**	**Mean (SD)**	116,856.1 (63,526.5)	84,607.1 (56,327.6)	44,416.7 (35,905.1)	0.06(9204.8 to 55292.9)	<0.0001(35801.6 to 109077.1)	0.04(1976.2 to 78404.7)
**SGOT (IU/L)**	**Mean (SD)**	88.5(111.6)	175.1(217.1)	1,259.1(1,320.5)	0.3(-255.8- 82.5)	<0.0001(-1439.6 to -901.5)	<0.0001(-1364.5 to -803.3)
	**>35 IU/L (%)**	44(66.7%)	33(78.6%)	11(91.7%)			
**SGPT (IU/L)**	**Mean (SD)**	54.9(58.2)	100.4(120.1)	479.3(397.1)	0.1(-103.1 to 12.2)	<0.0001(-516.1 to -332.7)	<0.0001(-474.6 to -283.3)
	**>45 IU/L (%)**	28(42.4%)	22(52.4%)	11(91.7%)			
**Alkaline phosphatase (IU/L)**	**Mean (SD)**	73.4(38.7)	89.1(70.5)	157.0(106.5)	0.2(-39.2 to 7.8)	<0.0001(-121.1 to -46.1)	0.001(-107.0 to -28.8)
	**>104 IU/L (%)**	7(10.6%)	9(21.4%)	7(58.3%)			
**Total bilirubin (mg/dl)**	**Mean (SD)**	0.5(0.3)	0.8(0.6)	1.2(1.4)	0.04(-0.4 to -0.006)	0.001(-1.0 to -0.2)	0.04(-0.8 to - 0.01)
	**>1.2mg/dl (%)**	1(1.5%)	7(16.7%)	3(25.0%)			
**Direct bilirubin (mg/dl)**	**Mean (SD)**	0.2(0.2)	0.3(0.5)	0.8(1.4)	0.2(-0.3 to 0.09)	<0.0001(-0.9 to -0.2)	0.004(-0.8 to-0.16)
	**>0.2 mg/dl (%)**	24(36.4%)	22(52.4%)	8(66.7%)			
**Serum albumin (g/dl)**	**Mean (SD)**	4.1(0.5)	3.9(0.6)	3.4(0.5)	0.02(0.05 to 0.47)	<0.0001(0.4 to 1.0)	0.008(0.13 to 0.83)
	**<3.5 g/dl (%)**	6(9.1%)	9(21.4%)	6(50.0%)			
**A/G ratio <1**	**Mean (SD)**	1.6(0.3)	1.4(0.3)	1.1(0.3)	0.02(0.04-0.48)	<0.0001(0.49- 1.20)	0.002(0.21- 0.96
	**N (%)**	1(1.6%)	3(7.3%)	3(27.3%)			

**Figure 1:**
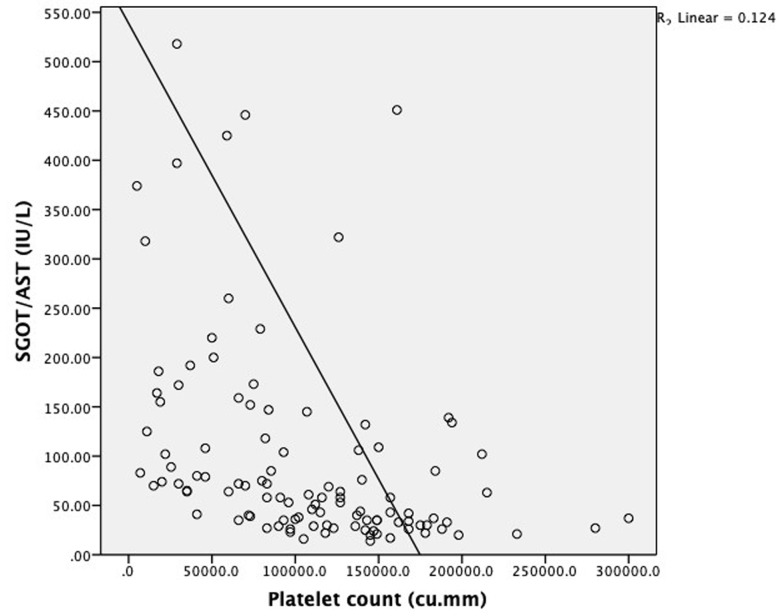
scatter diagram showing correlation between platelet count and SGOT in dengue patients

**Figure 2:**
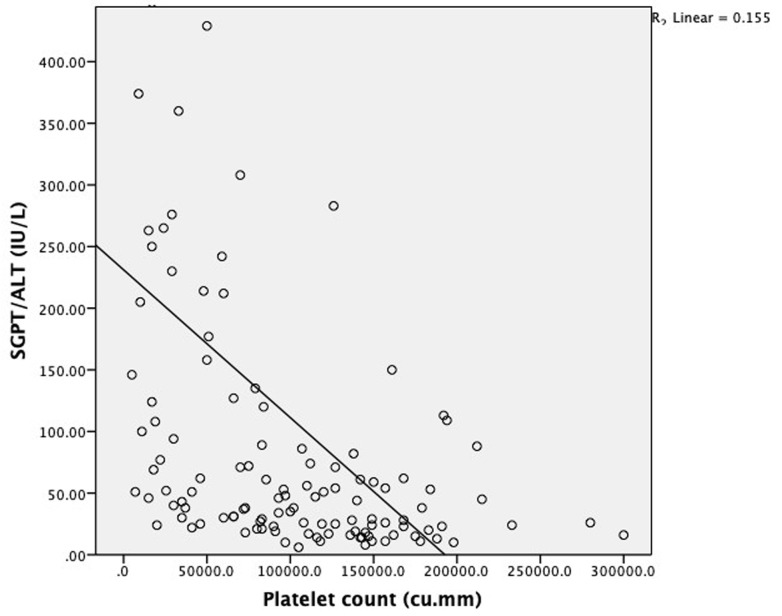
scatter diagram showing correlation between platelet count and SGPT in dengue patients

Elevated liver enzymes were seen in 75% of patients with abdominal pain, 90.9% of patients with persistent vomiting, 91.7% of patients with mucosal bleed, 100% of patients with lethargy/restlessness and 77.8% of patients with hepatomegaly >2 cm. Mean SGOT and SGPT levels were significantly elevated in patients with shock as compared to patients without shock [SGOT (1430(2033.4) vs. 184(325.7) IU/L: P<0.0001 respectively; SGPT (420.6(393.7) Vs. 99.9(169.5) IU/L: P<0.0001 respectively]. Mean hematocrit was significantly elevated in patients with elevated liver enzymes as compared to patients with normal levels of liver enzymes [Hematocrit (42.5(6.4) vs. 39.7(7.4) %; P=0.04 respectively]. Mean platelet count was significantly low in patients with elevated liver enzymes as compared to patients with normal levels of liver enzymes [Platelet count (81033.7(59256.8) vs. 147967.7(44726.9) cu.mm; P≤0.0001 respectively] Patients with elevated SGOT (93.8%) and SGPT (81.2%) had a higher incidence of bleeding manifestations. Serum glutamic-oxaloacetic transaminase and serum glutamic-pyruvic transaminase level increases with increase in dengue severity which is indicated by a fall in platelet count as they are negatively correlated with each other (P≤0.0001) as shown in Figure 1 and Figure 2 respectively.

## Discussion

In our study, we found that the prevalence of patients with dengue without warning signs (55%) was higher followed by dengue with warning signs (35%) and severe dengue (10%). Among these patients, 75 were males and the remaining 45 patients were females (M: F ratio-1.6: 1) which is similar to that reported in several other Asian studies, such as by Agarwal *et al*. [12], Ray *et al*. [13] and Wali *et al*. [14], where the M: F ratio was found to be 1.9: 1, 1: 0.57, and 2.5: 1 respectively. This is in contrast to a South American study which found the disease to be more common among females with a M: F ratio of 0.65: 1 [15]. This difference could be attributed to the lower self-reporting of the disease among women of Asian communities. Additionally, 7 out of 12 severe dengue patients in our study were females. Many other studies have also found that the disease has a more severe course in women which is attributed to the higher production of cytokines, increased permeability of the capillary bed and a competent immune response in females as compared to males [16,17]. The mean age of the patients in our study was 34.8 (15.1) years and 50.8% of the patients were aged between 18-30 years. A study by Gandhi *et al*. in Mangalore reported similar mean age among dengue patients [34.30±15.0 years] [18].

The broad spectrum of hepatic dysfunction in dengue varies from asymptomatic elevation of the transaminases to fatal fulminant hepatic failure. In our study, 74.2% of patients had elevated transaminases. SGOT was elevated in 73.3% of the patients and SGPT in 50.8% of the patients, which was similar to the findings of various other studies (Table 2) [17,19-26]. We found that the mean value of SGOT [235.9(550.6) IU/L] was more than double the mean value of SGPT [113.3(192) IU/L]. This is in stark contrast with other viral hepatitis which is characterized by higher SGPT values. Plausible mechanisms include release of SGOT from damaged erythrocytes, cardiac and skeletal muscle cells. This pattern of SGOT/SGPT derangement, along with the presence of thrombocytopenia and persistence of fever even after the appearance of icterus may hint towards dengue infection when the presentation and laboratory parameters mimic acute viral hepatitis [27]. The highest SGOT value found in our study population was 4942 IU/L which was more than 100 times the normal limit, while the highest SGPT value was 1306 IU/L which was nearly 30 times the upper limit. Both these patients had severe dengue and while the former patient died during the treatment, the other patient recovered within a short duration. Interestingly the first patient, despite having a stormy course, never developed any bleeding manifestations and the eventual cause of death was due to severe refractory hypotension but the second patient had severe bleeding manifestations at the time of presentation in the form of melena, thereby reinforcing the fact that SGPT continues to be the most specific marker of hepatic dysfunction. Mean alkaline phosphatase was significantly high in severe dengue as compared to the other groups in our study which was similar to the findings of the study done by Fadilah *et al*. [28].

**Table 2:** studies reporting hepatic dysfunction in dengue

Studies	Patients	Raised SGOT	Raised SGPT	SGOT>SGPT	Hypoalbuminemia	Raised bilirubin
**Our study**	120	73.3%	50.8%	+	17.5%	9.1%
Saha *et al*.	1226	52% (5 times normal was criteria)	50%	-	12.9%	16.9%
Souza *et al*.	1585	63.4%	45%	+	-	-
Shukla *et al*.	70	100%	91%	-	-	-
Kuo *et al*.	270	93.3%	82.2%	+	-	7.2%
Wong *et al*.	127	90.6%	71.7%	+ in 75.6%	16.5%	13.4%
Itha *et al*.	45	96%	96%	Equal	76%	30%
Parkash *et al*.	699	95%	86%	+	-	-
Trung *et al*.	644	97%	97%	+	-	1.7%
Lee *et al*.	690	86%	46%	-	-	-
Karoli *et al*.	138	92%	-	+	-	48%

We observed significant difference in mean values of liver function tests when compared across disease severity. Mean SGOT, SGPT ALP, total bilirubin and direct bilirubin was significantly higher and albumin levels were significantly low among severe dengue patients when compared with dengue with and without warning signs. Thus it can be concluded that elevation of the transaminases, total and direct bilirubin, ALP levels, hypoalbuminemia and A: G reversal are all markers of a more severe form of the disease. Similar conclusions have been drawn by Bandyopadhyay D *et al*. [27]. Tinambunan E *et al*. [29], Soni A *et al*. [30]. In our study, we found increase in SGOT and SGPT level with increase in dengue severity which is indicated by fall in platelet count as they are negatively correlated with each other.

Multiple mechanisms are responsible for liver injury in dengue such as direct viral cytopathic effects, immune mediated injury and hypo perfusion. Micro vesicular steatosis, hepatocellular necrosis, Kupffer cell hyperplasia and destruction, Councilman bodies, and inflammatory cell infiltrates are some of the changes observed in human post mortem studies. Immunohistochemistry studies have also shown infiltration of the hepatic acini with CD4+ and CD8+ T cells along with a higher expression of IFN-γ, thereby implicating the Th1 cells. Dengue is also known to cause microcirculatory dysfunction due to venular or sinusoidal endothelial injury which can cause hepatocyte ischemia, irrespective of the presence of hypotension [31]. In endemic areas, dengue can cause worsening of chronic liver disease resulting in acute on chronic liver failure.

Hypoalbuminemia as a part of the spectrum of hepatic dysfunction in dengue is not well studied. Saha *et al*. [11] and Wong *et al*. [21] have reported hypoalbuminemia among 12.9% and 16.5% of dengue patients respectively. We found hypoalbuminemia in 17.5% of the dengue patients. The contrast to our findings, Itha *et al*. [22] have reported higher prevalence of hypoalbuminemia (76%). In our study, we reported significantly low mean serum albumin in those with severe dengue. Amancio *et al*. [32] found that low serum albumin levels were more commonly associated with severe dengue and in those who had fatal dengue. Though dengue is an acute illness, hypoalbuminemia may be seen due to capillary leakage [16]. Accordingly, its correlation with the disease severity is justified. We also found that hypoalbuminemia was more commonly found in those patients who presented in shock. A: G ratio of less than 1 was significantly higher in patients with severe dengue (27.3%) than in those with dengue with warning signs (7.3%) and dengue without warning signs (1.6%). This may be explained by the difference in molecular size of albumin and globulin. Being a smaller molecule, albumin leaks out more easily than globulin during the early stages of the disease, thereby causing a reversal of the A: G ratio [33].

Jaundice is an ominous sign of poor prognosis in dengue and is multifactorial. Itha *et al*. [22] reported jaundice among 15% of their study population while several other studies have pegged the presence of elevated total bilirubin among dengue patients at 1.7%- 17% [20,24,26]. In our study, hyperbilirubinemia was seen in 9.1% of patients with dengue infection. Total bilirubin was elevated in only one patient of dengue without warning signs (1.5%), 16.7% of the patients with warning signs and nearly 25% of the patients with severe dengue. A severe dengue patient with predominant conjugated hyperbilirubinemia (5.2 mg/dl) had acute kidney injury (Serum creatinine 4.4 mg/dl) and also succumbed during the hospital stay. Presence of jaundice is a common finding in the pediatric population suffering from dengue as reported by Roy A *et al*. (60%) [34]. Summarily, hyperbilirubinemia, is not a common finding among the adult population as opposed to the pediatric population and the presence of the same can often mislead the physician. Hence dengue should always be considered as one of the differentials in all patients presenting with hepatomegaly and jaundice. Several studies have explored the relationship between hepatic dysfunction and the disease severity. However not many studies have addressed the relationship between the signs and symptoms of dengue and the laboratory parameters. The possibility of a sign or a symptom serving as a predictor of hepatic dysfunction is intriguing. A study by Karoli *et al*. [26] found that the most common symptoms found in dengue hepatitis were abdominal pain, vomiting and anorexia. In our study, we found that liver enzymes were elevated in 30 of the patients with persistent vomiting (90.9%) which was statistically significant. Transaminases were also elevated in 75% of the patients with pain abdomen and in all of the patients with hepatomegaly. Thus, the knowledge of such a relationship can help predict the presence of hepatitis in dengue patients during the initial presentation itself.

In our study, mean SGOT, SGPT and ALP levels were significantly elevated in patients with shock as compared to patients without shock. Though it has been postulated that hepatic dysfunction may be seen even in the absence of hypotension, due to microcirculatory dysfunction, the damage appears to be even more in the presence of shock [16]. Also, patients who presented with bleeding manifestations had significantly higher SGOT, SGPT, ALP and serum bilirubin when compared with patients with no bleeding manifestations. Hemoconcentration represented by higher mean hemoglobin and hematocrit was statistically significant in patients with elevated liver enzymes (p= 0.028 and p= 0.04 respectively).

In the study sample, we encountered two deaths; both were severe dengue female patients. Therefore, mortality from dengue does appear to be important, which can only be minimized by increasing the awareness about dengue specific signs and symptoms among people.

**Strengths of the study:** 1) first study conducted in adult population to estimate the prevalence of hepatic involvement in dengue patients based on 2009 modified categorization of WHO. 2) Study has addressed the knowledge gap in prevalence of hypoalbuminemia among dengue patients, values of liver enzymes among dengue patient with/without shock and bleeding manifestations. 3) Our study has addressed the relationship between the signs and symptoms of dengue and hepatic dysfunction

**Limitations of the study:** 1) the study sample is small, may be statistically less accurate compared to studies with a larger population and our study was a retrospective study. 2) The patients were selected from a tertiary care center which usually tends to see a clustering of more severe cases as the less severe ones may be treated on out-patient basis. Hence the results of the study may not be an accurate representation of the entire population. 3) Liver biopsy which is a definitive diagnostic test of dengue hepatitis was not done due to financial and ethical reasons.

## Conclusion

Liver involvement in the form of elevated transaminases was found in 74.2% dengue patients. Serum glutamic-oxaloacetic transaminase and serum glutamic-pyruvic transaminase level increases with increase in dengue severity which is indicated by fall in platelet count as they are negatively correlated with each other. Serum glutamic-oxaloacetic transaminase was elevated more than serum glutamic-pyruvic transaminase in dengue patients. Liver damage is one of the common complications of dengue and transaminitis, hypoalbuminemia and reversal of A: G ratio should be used as biochemical markers in dengue patients to detect and monitor hepatic dysfunction.

### What is known about this topic

- *Hepatic dysfunction is commonly seen in dengue and is related to the disease severity;*
- *It mainly occurs in the form of elevation of the transaminases*.

### What this study adds

- *The importance of identifying specific dengue warning signs (such as persistent vomiting, pain abdomen, hepatomegaly, bleeding manifestations and shock) that are predictive of hepatic dysfunction;*
- *While SGOT and SGPT are commonly evaluated in dengue patients, serum albumin levels and A: G ratio are usually neglected. Our study shows that hypoalbuminemia and A: G ratio <1 are markers of disease severity. Hence, they need to be included in the initial workup of dengue patients for prognostication*.
